# Progress on genome-wide association studies and selection signature analysis for agronomic traits in Chinese sheep and goats: review

**DOI:** 10.5713/ab.25.0465

**Published:** 2025-10-22

**Authors:** Mingxuan Han, Youjun Rong, Bingjie Ma, Xinle Wang, Xiaofang Ao, Fangzheng Shang, Rui Su, Ruijun Wang, Yanjun Zhang

**Affiliations:** 1 College of Animal Science, Inner Mongolia Agricultural University, Hohhot, China; 2 Key Laboratory of Mutton Sheep Genetics and Breeding, Ministry of Agriculture, Hohhot, China; 3 Key Laboratory of Goat and Sheep Genetics, Breeding and Reproduction in Inner Mongolia Autonomous Region, Hohhot, China

**Keywords:** Agronomic Traits, China, Genome Wide Association Studies, Goat, Selection Signature, Sheep

## Abstract

Sheep and goats, among the earliest domesticated animals, hold significant historical importance in Chinese animal husbandry. The rapid advancement of modern biotechnology has rendered genome wide association studies (GWAS) and selection signature analyses have become indispensable tools in livestock genetic research. This review summarizes recent progress in the application of these methods to sheep and goat breeding in China, analyzes existing challenges in the research, and suggests potential research strategies. GWAS and selection signature analyses have identified key genomic regions associated with agronomically important traits. Genes such as *LHX2*, *FGF12* and *Notch3* are associated with hair follicle growth and development, while *RBM11*, *SMARCA5* and *GAB1* are linked to body size in sheep. Additionally, *BMPRIB* has been identified as a determinant of reproductive performance and growth rate in both sheep and goats. Despite progress, several challenges remain, including incomplete reference genomes, insufficient phenotypic data, inadequate algorithms, and a lack of functional validation and practical application of findings. Future work must prioritize genomic refinement, the integration of multi-omics, and the development of algorithm. Enhanced international collaboration is crucial to deciphering the genetic basis of key traits, thereby advancing global industry. These initiatives will enable highly precise breeding strategies. Through precise identification and selection of individuals with desirable genetic traits, breeders can significantly enhance the efficiency of genetic improvement. Using Hua sheep 50k chip as an example, it effectively identifies individuals with superior reproductive genes (such as *BMPRIB*) at an early stage, increases the lambing rate by 27.7%, accelerates the propagation of high-quality breeding groups, provides a large number of breeding sheep with excellent genetic traits for the global sheep industry, and improve the overall quality of germplasm. This will ultimately enhance the sustainable development of global sheep and goat production.

## INTRODUCTION

Comparative genomics shows that sheep and goats were domesticated in the Middle East about 10,500 years ago. Later humans spread domesticated sheep around the world, marking one of the earliest cases of livestock domestication [1]. Following domestication, goats gradually spread into Europe through the Danubian and Mediterranean corridors. The Eurasian steppe belt provided an important passage for goats to spread to Asia [2]. China has rich sheep and goat varieties and diverse regional types, and its rich genetic diversity is an important part of biological genetic resources. Breed development began in the late 1700s, resulting in remarkable genetic diversity and improvements in livestock all over the world [3]. At the beginning of the twentieth century, the emergence of molecular genetic techniques has allowed for even more genetic advancements and led to a significant increase in productivity in all major livestock species [4]. Concurrent improvements in breeding value estimation, computing methods, and selection accuracy, initially through quantitative genetic approaches and more recently incorporating genomic tools, have further enhanced the rate of genetic improvement over time [5]. The advent of molecular genetics in 1970s opened up new possibilities for developing livestock breeding programs through DNA markers to identify genes or genomic regions that regulate traits of interest [3]. In the 1980s, the concept of a quantitative trait locus (QTL) has been proposed in the field of animal breeding, which assumed that genes with related functions were often clustered in the genome to control biological traits. Since then, the QTLs in association with genes or chromosome segments of interest have been widely studied to delineate complex animal traits [6]. In addition, the emergence of genomics as a discipline led to the concept of marker assisted selection (MAS), in which genetic variants and genes that influence agriculturally important traits would be identified and used to further increase genetic response [7]. Collaborations were established to maximize the relatively meager resources available to animal genetics in the early 1990s. At the beginning of the 21st century, the completion of the human genome project also marked a new era of genome research, and at the same time, livestock genome breeding also entered a new revolution. Genome sequencing was greatly democratized by the development of next generation sequencing (NGS) technologies [8], high-density SNP arrays, advanced statistical and bioinformatics tools have greatly improved our ability to detect the genomic regions under selection in livestock species. Genome research in livestock species also advances our knowledge of chromosomal evolution and the human genome [9]. Genomic technologies like sequencing and gene editing, along with advanced computing, accelerate genetic progress to meet growing protein demand sustainably [10]. China’s sheep and goat industries have also experienced rapid development due to these technologies. For instance, with the help of the 50k chip for Hu sheep independently developed by China, the lambing rate has increased by 27.7%. This chip can complete selection within 1–3 days after lamb birth, replacing the traditional 12 months selection cycle, significantly shortening breeding time and increasing economic benefits. The identification of these effective loci relies on genome wide association studies (GWAS) and selective trait analysis, which have become indispensable tools in genetic breeding research.

**Figure 1:**
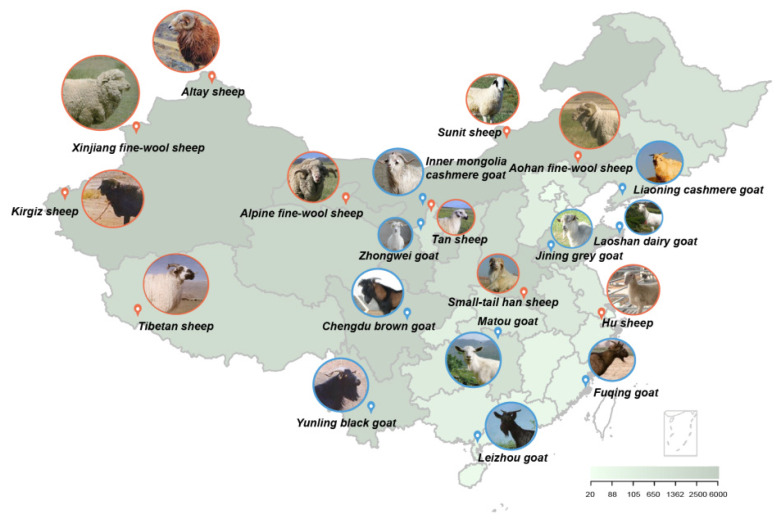
Distribution of sheep and goats germplasm resources in China. Blue represents goats, orange represents sheep, the gradient color represents the number of sheep and goats in each province of China.

According to the National Variety list of Livestock and Poultry genetic Resources, by the end of 2025, there are currently 110 sheep breeds in China, including 58 local breeds, 38 cultivated breeds, 14 introduced varieties and 90 goat breeds, including 69 local breeds, 15 cultivated breeds and 6 introduced varieties (Figure 1). Due to long term insufficient scientific understanding and evaluation of their value, China’s local sheep and goat populations have dropped sharply. Current genomic studies mostly focus on high-value breeds like fine-wool sheep or Inner Mongolia Cashmere Goat, while breeds such as Yunling and Leizhou goats lack genetic research, with scarce related data. Different teams often get inconsistent results on the same traits due to varied sample collection, microarray versions and analytical methods, making it hard to reach conclusions. Moreover, over 60% of GWAS only identify candidate genes without laboratory validation of their functions.

This review aims to showcase the latest advancements in GWAS and selection signature analyses for Chinese sheep and goat populations. In this review, we first explore the development of genomic technologies in livestock and the progress of genomic resources for sheep and goats, with a focus on reviewing the research advancements of GWAS and selective signature analysis in important economic traits of Chinese sheep and goats. Finally, we analyze the major challenges faced by emerging countries represented by China in the genomic breeding process of sheep and goats, and look forward to future trends in this field. Overall, this issue helps better understand on how to leverage genetic variation through genomics to improve sheep and goat populations.

## GENOME RESOURCES FOR SHEEP AND GOAT

Back in early 2007, a major leap in sequencing technology allowed scientists to start doing de novo sequencing on sheep and goats. This breakthrough opened doors for creating detailed SNP chips and assembling whole genomes. The main benefits of these high-density breeding chips are their broad coverage, accurate detection, and quick genotyping, making the breeding process faster and more efficient. When we talk about genome assembly, we’re referring to the process of piecing together DNA sequences to form complete, continuous chromosome-level genomes. This gives us a full picture of an animal’s genetic makeup and forms the foundation for all kinds of genetic research and breeding work.

In 2010, the Sheep Genomics Consortium successfully developed an upgraded version of the Virtual Sheep Genome (VSG2). By leveraging data from other mammalian genomes, the Consortium added over 190,000 additional data points. Concurrently, the International Sheep Genome Consortium (ISGC) forged a partnership with Illumina to create the OvineSNP50 BeadChip. This comprehensive whole genome SNP chip for sheep was, at that time, the most extensive whole genome genotyping array available. It greatly expedited the exploration of genetic variations across a wide range of sheep breeds, contributing to more informed breeding decisions [11]. Oar_v1.0, the initial major iteration of the sheep reference genome, was predominantly constructed using the Sanger sequencing method. This early DNA sequencing technology was renowned for its high accuracy. However, due to the constraints of sequencing technology and assembly algorithms prevalent at that time, Oar_v1.0 exhibited relatively low contiguity and completeness. The genome assembly was marred by numerous gaps and unresolved issues. With the advent of more advanced bioinformatics tools, Oar_v1.0 underwent successive updates. In 2015, Oar_v4.0 was introduced. Representing the pinnacle of technology at the time, Oar_v4.0 marked a substantial improvement over its predecessors. It offered more comprehensive genome coverage and greater accuracy. This enabled researchers to conduct more in depth analyses of the genetic structure and evolutionary relationships of sheep, and to carry out diverse genome based application studies. Currently, ARS-UI_Ramb_v3.0 is another widely utilized sheep reference genome among researchers. In 2024, the CAU_Oori_1.0 sheep reference genome, derived from the Asiatic mouflon, was successfully assembled. Based on this genome version, Yang et al [12] characterized and compared the structural variation (SV) landscape of sheep by analyzing aspects including the chromosomal distribution of SVs and their associations with genes, QTL, and transposable elements (TEs). This study represents the largest and most comprehensive investigation to date on the convergent evolution of SVs across different genera of mammals. Subsequently, Luo et al [13] augmented ARS-UI_Ramb_v3.0 by adding 220.05 Mb of previously uncharacterized regions. They also identified 754 new genes, presenting a gapless telomere to telomere genome of 2.85 Gb for rams (T2T-sheep1.0). This genome encompasses all autosomes, along with the X and Y chromosomes. T2T-sheep1.0 boasts a base accuracy exceeding 99.999%. It rectifies several structural errors present in previous reference assemblies and enhances the detection of SVs within repetitive sequences (Table 1). Telomere-to-Telomere (T2T) gapless assembly is a complete genome that fully covers all chromosomal regions without missing fragments or assembly errors. It marks the transition of genome assembly from “fragmented coverage” to the stage of “full-sequence precise analysis” and holds revolutionary significance for life science research.

**Figure 2:**
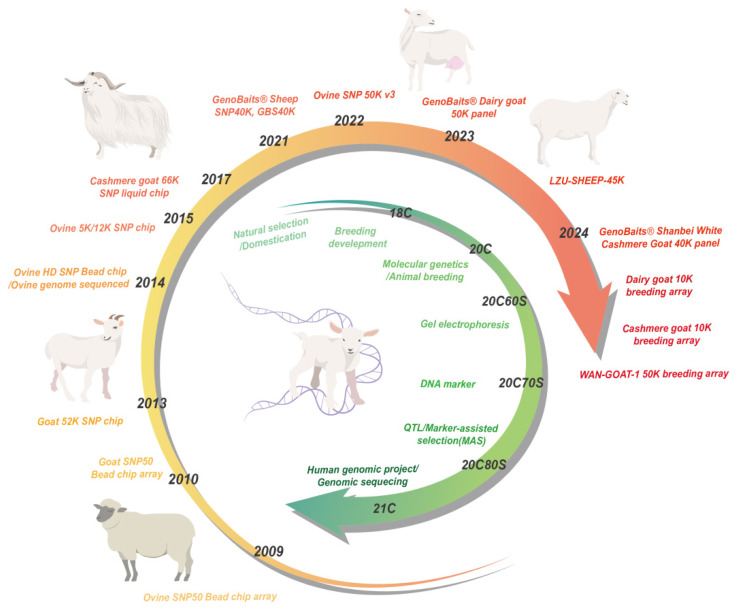
The timeline of genetic improvement of livestock (green) and the sheep and goat SNP chip (orange).

In 2013, the inaugural goat reference genome, CHIR_1.0, was assembled through short read de novo sequencing. The genomic material was sourced from a Yunnan doe. Subsequently, in 2015, two revisions were carried out, and the refined data were leveraged to develop the first 50K goat SNP chip [14]. During the development of this 50K goat SNP chip, whole genome sequencing data from multiple goat breeds had already been integrated. In 2017, the ARS1 reference genome was launched. This genome assembly harnessed advanced sequencing technologies, including Illumina short read sequencing and PacBio long read sequencing, in conjunction with optical mapping data. As a result, it demonstrated enhanced contiguity and completeness compared to previous versions. This technological leap significantly propelled research in goat genomics [15]. In 2017, a targeted enrichment strategy grounded in SHS enabled the successful development of a 66K SNP chip specifically for cashmere goats [6]. In 2021, Li et al [16] introduced a highly accurate de novo genome assembly for Saanen goats (Saanen_v1). This assembly demonstrated remarkable completeness, rectifying eight putative large scale assembly errors (each spanning from 1 to approximately 7 Mb) present in the ARS1 assembly. Moreover, it provided the initial estimate of the substitution rate of the Y chromosome in this ruminant species. In 2023, Guan et al [17] developed a 54K whole-genome liquid SNP chip for dairy goats, which serves as a potentially valuable and cost effective tool for GWAS studies on milk composition traits. Furthermore, the LZU-SHEEP-45K Hu sheep breeding chip, along with 50K, 20K, and 5K liquid phase gene chips for dairy goats, and the WAN-GOAT-1 50K breeding array, were successfully developed. These achievements established a genomic breeding technology system with full independent intellectual property rights, tailored to the breeding of Chinese sheep and dairy goats. This milestone represented a significant breakthrough in the field of sheep and dairy goat genomics breeding, placing China at the forefront alongside international counterparts [18]. In 2024, Wang et al [18] generated an almost complete genome of Inner Mongolia cashmere goats using PacBio HiFi, ONT ultra long reads, and Hi-C technologies. Concurrently, Wu et al [19] assembled a telomere to telomere gap free cashmere goat genome (T2T-goat1.0). With a base accuracy surpassing 99.999%, this assembly effectively corrected numerous structural and base level errors across the genome in previous assemblies. It also added 288.5 Mb of previously uncharacterized regions and 446 newly assembled genes to the reference genome, furnishing invaluable resources for goat genetics and genomics research. Additionally, a 5K liquid chip was developed, enabling accurate identification of three breeds: Inner Mongolia white cashmere goats, Hanshan white cashmere goats, and Ujimqin white cashmere goats (Figure 2) [20].

As new SNP chips and genome assembly technologies have advanced, China has built a comprehensive genomic breeding system personalized for Chinese sheep and dairy goats. By shortening the generation interval, improving selection accuracy, and expanding selection scale, it multiplies the rate of genetic benefit accumulation. Simultaneously, it enables breeding companies to transition from “long cycles” to “agile operations”, and farmers to shift from “blind practices” to “precision-driven approaches”, ultimately driving industrial upgrading across the sector. Still, current goat and sheep reference genomes face challenges with assembly quality, accuracy, and understanding complex regions. For example, although T2T-goat1.0 fixed some errors, other structural issues might still exist. Also, most reference genomes are based on only a few breeds or individual animals, which means they don’t fully capture the genetic diversity of all goats and sheep. This can result in missing some important genetic traits. Despite these obstacles, current technologies have been essential in advancing research related to wool and cashmere growth, body development, muscle growth, milk production, reproduction, and how these animals adapt to their environment.

## PROGRESS IN GENOMIC RESEARCH

### Cashmere and wool trait

China is a globally significant producer and consumer of cashmere and wool, with approximately 70% of the world’s cashmere originating from China, and its quality surpasses that of other countries. By leveraging native breeds such as cashmere goats (Inner Mongolia Cashmere Goat, Liaoning Cashmere Goat), fine-wool sheep (Chinese Merino Sheep, Northeastern Fine-wool Sheep), and semi-fine-wool sheep. China has established a raw material supply system tailored to domestic textile demands. As core economic products of goat and sheep industries, the quality (fineness, length, color) and yield of cashmere and wool directly determine industrial value. However, the formation of cashmere and wool results from the coordinated action of multiple physiological pathways, with each trait being jointly regulated by hundreds or even thousands of genes. Although major genes contribute more significantly to traits, their signals are easily obscured by the “genetic background noise” from numerous minor genes. Consequently, traditional breeding methods alone struggle to elucidate their regulatory mechanisms. Whole GWAS and selection signal analysis serve as critical technological bridges for uncovering regulatory mechanisms governing economic traits in cashmere and wool production.

#### Selection signatures analysis

Sequence heterozygosity and divergence were analyzed between the well known Inner Mongolia Cashmere goat breed and other goat breeds. As a result, candidate genes associated with cashmere traits were identified. Among these, *LHX2* plays a role in the development of secondary hair follicles in cashmere goats. *FGF9* and *Wnt2* potentially account for the cyclic growth of cashmere fibers. Additionally, the coat color gene *MC1R*, which controls white coat color, and *FGF5*, which regulates hair length, were mapped [21]. In another study, *CHRM2*, *SDC1*, *ITCH*, and *FGF12* were found to be associated with wool growth [6]. Ma et al [22] conducted research on whole genome copy number variation (CNV) in Tan sheep. Their findings revealed that the *DLX3* gene within the CNV region is linked to the characteristic coiled wool phenotype. Jin et al [23] employed Fst and XP-EHH test statistics to identify the genomic features related to cashmere traits in Chinese goats (Inner Mongolia and Liaoning cashmere goats, as well as Huanghuai goats). Their results demonstrated that the *WNT10A* and *CSN3* genes are significantly associated with cashmere traits in goats. Lv et al [24] conducted whole genome sequencing on seven wild species and 158 distinct domestic sheep populations. Through paired comparisons of hairy, coarse, medium, and fine wool sheep populations, they employed XP-CLR statistics and π ratio statistics to explore potential selection signals within the genomes. This research revealed a significant role of the *IRF2BP2* gene in relation to wool fiber diameter. Combining selective sweep analysis with transcriptome data, it was discovered that expression differences between *KRT* and *KAP* could directly account for the phenotypic disparities in cashmere fineness between two breeds [25]. Wang et al [26] utilized whole genome resequencing data to analyze the genomic features of artificially selected cashmere goats. By integrating transcriptome data and focusing on genes involved in the regulation of cashmere traits, they identified type IV collagen family genes (*COL4A2*/*4*/*5* and *COL6A5*/*6*) and integrin family genes (*ITGA2*/*4*/*9* and *ITGB8*) as potential key candidates for the formation and development of cashmere. ROHs analysis indicated that *FGF5*, *DVL3*, *NRAS*, and *KIT* were associated with fiber length or color [27]. To elucidate the genetic basis of cashmere traits, Wu carried out cross population selection analysis between cashmere and non cashmere goat populations. This analysis identified selection signals associated with cashmere traits, including *KRT*, *FGF5*, *EDA2R*, *ABCC4*, *ASIP*, *FGF6*, and *MCOLN3*. These findings were consistent with those of Cai and Luo [19]. Based on the latest sheep reference genome, Luo et al [13] determined that *FOXQ1* exhibited a strong selection signal in relation to wool fineness.

Chinese native breeds exhibit remarkable diversity in wool color. Through whole genome sequencing and selection signal analysis, researchers have uncovered several candidate genes and regulatory pathways associated with hair color. In multiple goat breeds, genomic selection signal analysis has pinpointed key genes including *KITLG*, *IRF4*, and *MC1R* [28,29]. Among these, the mutant form of the *MC1R* gene shows a significant association with the black hair color phenotype. Its selection signal has been detected in both Tibetan goats and Bohuai goats [29,30]. Regional adaptation studies indicate that, in Bohuai goats (a cross between Boer and Huai goats), a selection region on chromosome 7 is linked to coat color, potentially associated with artificial - selection targets such as growth rate and coat color [29]. *ASIP*, *KITLG*, *HTT*, *GNA11*, and *OSTM1* have also been identified as crucial genes related to goat coat color [21]. Moreover, the grey coat characteristic of Tibetan goats may be associated with the plateau adaptation gene *EPAS1*, suggesting a co-evolution between coat color and environmental adaptation, the roles of *ASIP* and *LITLG* were validated in Guo’s research [28]. Based on population structure analysis and Fst values, significant differences in allele frequencies at the c.-253G>A site within the 5’UTR of *FGF5* were detected between cashmere goats and short haired goat breeds. This polymorphism is likely a natural causal variant contributing to the long haired phenotype in cashmere goats [31]. Another study demonstrated that *TCF25* influences the black middle dorsal stripe in Youzhou black goats [32]. The iHS is utilized to analyze high frequency SNPs within ROH regions in relation to genes determining coat color. Through this approach, genes *OCA2* and *MLPH*, associated with coat color, have been identified [33]. Guan et al [17] detected a significant signal associated with hair color at 31.52–35.02 Mb on chromosome 8, and the *TYRP1* gene was found to be located in this genomic region [33]. Zhang et al [34] employed whole genome resequencing data to conduct a comparative analysis of sheep populations from different breeds using population differentiation indices, nucleotide diversity ratios (Fst and θπ), and extended haplotype purity between populations (XP-EHH). This analysis revealed *ABCD4*, *VSX2*, *ITCH*, *NNT*, *POLA1*, *IGF1R*, *HOXA10* and *DAO*, which are associated with the formation of white wool. Analysis of selection signatures using multiple methods (Fst, θπ, and Tajima’s D) has identified genes such as *MC1R*, *MLPH*, *SPIRE2*, *RAB17*, *SMARCA4*, *IRF4*, *CAV1*, *USP7*, *TP53*, *MYO6*, *MITF*, *MC2R*, *TET2*, *NF1*, *JAK1*, and *GABRR1* that affect melanocyte formation, melanin synthesis, and melanin transport. Conversely, genes like *REST*, *POU2F1*, *ADCY10*, *CCNB1*, *EP300*, *BRD4*, *GLI3*, and *SDHA* influence the formation of white wool. Common genes such as *PPP2R5E*, *CCDC39*, and *CAMK2D* play multiple regulatory roles in both black and white wool color, providing valuable insights for genetic improvement and selection in Tibetan sheep [29].

#### Genome-wide association study

GWAS was conducted to explore the fiber length, fiber diameter, and cashmere yield of Inner Mongolia Cashmere goats, utilizing the Illumina GoatSNP52K Beadchip panel. The study revealed that genes such as *FGF12*, *SEMA3D*, *EVPL*, *SOX5*, *Notch3*, *BMPR1B*, and *CCNA2* have direct functional associations with fibroblasts and hair follicle stem cells. These genes play crucial roles in the growth and development of hair follicles [35,36]. Ma et al [37] identified 208 significant SNPs associated with birth wool length. This discovery implicated *RAD50*, *MACROD2*, *SAMD5*, *SASH1*, and *SPTLC3* as potential candidate genes for this trait. Notably, Ma et al [37] also confirmed the significant association of the *MC1R* gene with the head coat color. Zhao et al [38] employed GWAS to investigate potential genes (*UBE2E3*, *TNFSF4*, and *RHPN2*) within the genomic region of economic significance in Chinese fine wool sheep. In another study by Zhao et al [39], a whole-genome sequencing approach was applied to 460 fine wool sheep breeds to identify potential genes associated with wool fiber attributes of economic importance. These studies showed that *PLA2R1*, *FAM46A*, and *PLCE1* are associated with fiber length. Zhao et al [40] integrated GWAS signals and RNA-seq data and found that *BNC1* is associated with the average wool fiber diameter and hair color and is specifically expressed in skin tissue. The GGP_Goat_70K SNP chip was utilized to genotype northwest Tibetan white cashmere goats. As a result, genes such as *CLNS1A*, *CCSER1*, *RPS6KC1*, *PRLR*, *KCNRG*, *KCNK9*, and *CLYBL* were identified as important candidate genes for wool traits [41].

### Growth trait

Goat and sheep as economically important animal breeds, their growth traits and body weight directly affect the efficiency of livestock production. In recent years, with the rapid development of genomics technology, Chinese scholars have made remarkable progress in analyzing the genetic mechanisms of growth traits in goats and sheep. Through GWAS, selection signature detection, SV and CNV analysis, a number of candidate genes and genomic regions related to growth rate, body weight and body size characteristics have been identified, providing a theoretical basis for molecular marker assisted breeding [42].

#### Selection signatures analysis

Selection signature analysis pinpointed a strongly selected genomic region on chromosome 22, spanning positions 51,425,001–51,500,000. The *CYP2E1* gene, located within this region, exhibits a strong association with growth traits [43]. Wang et al [21] identified *TBX15*, *DGCR8*, *CDC25A*, and *RDH16* as key genes influencing body size across multiple goat breeds. Through ROH and selection signature analysis, *TNPO2*, *IFT80*, *UCP2*, *UCP3*, *GHRHR*, *SIM1*, *CCM2L*, *CTNNA3*, and *CTNNA1* were detected to play roles in the growth and development of Youzhou black goats [32]. *HMGA2* and *GJA3* have been associated with goat growth, while *POMC* has been linked to goat body weight [33,44]. Whole genome resequencing of Hu sheep elucidated the genetic basis for their rapid growth. This analysis identified significant selection signals for *GPR35* and *NAV1*, which are associated with immunity and growth [45]. Analysis of selection signals and SVs further unveiled artificially selected genomic imprints. For instance, on chromosome 7 of BoHuai goats, selective regions related to growth rate were identified. These regions involve genes such as *BMPR1B* and *GHR* [30].

#### Genome-wide association study

An excellent GWAS study showed *RBM11*, *SMARCA5*, and *GAB1* were associated with body size in Hulun Buir sheep [46]. To gain further insights into genetic factors influencing body characteristics, Hu sheep parents and their offspring were genotyped. GWAS were conducted on multiple traits, including body weight, body height, chest circumference, body length, tail length and tail width. As a result, *KITLG* and *CADM2* were found to be closely related to body height, *MCTP1* and *COL4A6* were significantly associated with chest circumference, and *CAPN6* was strongly linked to body weight [11,47]. Similarly, GWAS on growth indicators of Zhongwei goats at different growth stages identified *ADIPOQ*, *CCDD39*, *PTPRT*, *ZNF215*, *VRTN*, *ABCD4*, *DLST*, *ADAMTS2*, *ROBO1*, *AKAP13*, *AQP1*, *SOX2* and *AHSG* as candidate genes potentially regulating growth traits [48]. Employing the same approach, *GFB2*, *BAG3*, *ZEB2*, *KCNJ12*, *MIF*, *MAP2K3*, *HACD3* and *MEGF11* were determined to affect the growth traits in Inner Mongolia cashmere goats [49]. In addition, *FAM184B*, *NCAPG*, *MACF1*, *ANKRD44*, *DCAF16*, *FUK*, *LCORL* and *SYN3* have been reported as candidate genes influencing the 14th month live weight in sheep [50]. In cashmere goats, *RUNX1T1*, *ERBIN*, *MYO15B*, *NT5C*, *GRB2*, *ITGB4*, and *GALK* were identified to impact the primary weight and weaning weight [51]. Moreover, research on the 8 month body weight of Hu sheep has shown that genes like *MAP3K1* and *MEF2C* modulate the cell cycle, thereby influencing the growth rate [52]. Similarly, GWAS of Tibetan Plateau type Tibetan sheep has identified genes including *HYDIN* and *IFI35*. These genes are associated with both plateau adaptation and muscle development [53,54]. In addition, GWAS investigations in Tibetan sheep have revealed that *FOSL2*, *KCND2*, and *TGFBI* are linked to body weight, body height, and chest circumference [55].

### Reproductive performance

The reproductive trait holds significant economic value for sheep production, regulated by heredity, hormone, environment, and managing factors. Previous studies have demonstrated the pivotal role of genetic factors in determining the reproductive performances of sheep and goats [56]. Reproductive characteristics have a direct impact on the productivity and economic benefits of the sheep industry [57]. Litter size is an important reproductive trait in sheep. Increasing litter size can rapidly expand the number of sheep, providing more high quality lamb, wool and dairy products to meet market demand. Using GWAS and selection signatures analysis to explore the high reproductive traits of sheep and goats help to accurately analyze the genetic mechanisms, and further facilitates molecular marker-assisted selection breeding, thus promoting the conservation and utilization of sheep breed resources.

#### Selection signatures analysis

In an earlier study, breeding relevant genes such as *CCNB2*, *AR*, *ADCY1*, *DNMT3B*, *SMAD2*, *AMHR2*, *ERBB2*, *FGFR1*, *MAP3K12*, and *THEM4* were specifically identified in the high reproductive Laoshan goat group. Conversely, *KDM6A*, *TENM1*, *SWI5*, and *CYM* were uniquely detected in the low reproductive Laoshan goat group. Additionally, genes including *SYCP2*, *SOX5*, and *POU3F4* were located within the selection windows for both high and low fecundity [58]. Through ZHp and RT-PCR Fst mixed pool analysis, 96 candidate genes potentially influencing goat fecundity were recognized. These genes encompass *NR6A1*, *STK3*, *IGF2BP2*, *AR*, *HMGA2*, *NPTX1*, *ANKRD17*, *DPYD*, *CLRB*, *PPP3CA*, *PLCB1*, *STK3*, and *HMGA2* [59]. Similarly, in Jining grey goats renowned for high litter sizes, *STIM1*, *SPIRE2*, *KIT*, *KCNH7*, *KMT2E*, *PAK1*, *PRKAA1*, and *SMAD9* exhibited strong selection signals [60,61]. Based on CNV differences among goats with varying litter sizes, Zhang et al [62] demonstrated that prolactin related proteins 1 and 6 (*PRP1* and *PRP6*) serve as crucial regulators of the reproductive process [62]. Employing 50K Illumina Bead Chips, the ROH and selection signals of six Chinese goat populations were characterized. As a result, *MARF1*, *SYCP2*, *TMEM200C*, *SF1*, *ADCY1*, and *BMP5* were pinpointed as candidate genes associated with goat fecundity [63]. Furthermore, CNV comparison and selection sweep analysis indicated that the *CBLB* gene might impact the litter size of Dazu black goats via SV. Moreover, Chr1_50215501 could potentially be utilized as an effective genetic marker for MAS breeding [64]. Comparative genomic studies have determined the genetic convergence of fecundity between goats and sheep. Consequently, *CHST11* and *SDCCAG8* are regarded as strong candidates influencing litter size in goats. *METTL15* and *MEI1* are characterized by selection in the genomic region of Guizhou black goat and is closely related to its fertility [65]. Across different goat breeds, *CCSER1*, *PDGFRB*, *IFT88*, *LRP1B*, *STAG*, *SDCCAG8*, *GRID2*, *ZNF276*, *TCF25*, *SPIRE2*, *TSHR*, *TSHB*, *PTGS2*, *ESR2*, *ATP5E*, *EPHA6*, and *CDKAL1* were all confirmed to exhibit selective characteristics and are associated with reproduction [33,44,66–68]. In the exploration of the association between kidding number in cloud based black goats and whole genome SVs, the pivotal roles of *BMPR1B* and *BMPRIB* in determining the reproductive performance of goats and sheep were identified. Specifically, *BMPR1B* was found to be more prominently expressed in the ovary, corpus luteum, endometrium, and cervix of sheep compared to other tissues. In goats, its expression in follicles is higher than in muscle and skin tissues [12].

### Milk production trait

Goat milk is a globally significant dairy product. Its production has been growing steadily due to its unique nutritional composition and adaptability. Goat milk contains bioactive compounds, is more digestible and less allergenic than cow milk, making it suitable for lactose intolerant consumers. The differences in physicochemical properties, like fatty acid composition and protein structure between goat and cow milk, endow goat milk with enhanced functional properties. In developed countries, goat milk also shows advantages in processing technology, product diversity, and health benefits [69]. Thus, improving goat milk quality and composition is great for practical significance and market value. In dairy goat research, GWAS and selection signature scanning have identified genomic regions and candidate genes related to economically important traits, such as milk yield and composition [70].

#### Selection signatures analysis

Xiong et al [67] compared the genetic diversity and selection characteristics in dairy goats, identifying several candidate genes. Specifically, *GHR*, *DGAT2*, *ELF5*, *GLYCAM1*, *ACSBG2*, and *ACSS2* were associated with milk production traits. Notably, *GHR*, a strongly associated gene, was also identified as closely related to milk production in Zhao’s study. Moreover, *STK3* and *PRELID3B* may serve as important candidate genes [68]. *DGAT1*, a key gene in milk production, was found to overlap with a novel CNV in goats. This overlap was significantly associated with milk production traits. In Xinong Sannen dairy goats, those with copy number loss exhibited high freezing point suppression and milk solid non fat content [71]. Through Fst and π ratio analysis, Ni et al [72] determined that *ANPEP*, *ADRA1A*, and *PRKG1* were associated with milk production in Guanzhong dairy goats. Intriguingly, *ADRA1A* was similarly identified in the Selionova’s study as being associated with milk production [73]. Zhao et al [74] employed SNP and Indel to identify population selection signals in dairy and non dairy goats. As a result, genes associated with milk production traits, such as *MPP7*, *PRPF6*, *DNAJC5*, *TPD52L2*, *HNF4G*, *LAMA3*, *FAM13A*, and *EPHA5*, were identified. By conducting a longitudinal GWAS on milk producing traits in 298 dairy goats, additional genes including *TTC39C*, *LAMA3*, *ANKRD29*, *NPC1*, *RIOK3*, *TMEM241*, *CABLES1*, and *RBBP8* were discovered. Transcriptome analysis of different lactating breast tissues revealed dynamic expression changes of *LAMA3*. Specifically, the average milk yield of TT homozygotes for the SNP_24_33,185,872 in this gene was significantly higher than that of TC and CC genotypes.

### Tail type trait

Sheep can be classified into three types based on their tail characteristics: fat-tailed, fat-hipped, and thin-tailed. The fat stored in the tail or hips serves as an energy reserve, supporting migration and survival during cold winters to adapt to harsh environments [75] and in times of material scarcity, people also have access to valuable edible fats. Exploring candidate genes affecting tail shape in sheep can help to accurately locate key genetic factors and analyze the tail formation mechanism, which provides strong support for sheep breed improvement, production performance optimization and genetic resource protection.

#### Genome-wide association study

Chinese indigenous sheep are categorized into fat tailed, fat rumped, and thin tailed types, with the Large tailed Han, Altay, and Tibetan breeds serving as representatives. Zhu employed high density ovine 600K SNP arrays to detect CNVs and SNPs. As a result, the first high resolution CNV map for these breeds was constructed. In Large tailed Han, Altay, and Tibetan sheep, 371, 301, and 66 CNV regions were identified respectively. Genes such as *PPARA*, *RXRA*, *KLF11*, *ADD1*, *FASN*, *PPP1CA*, *PDGFA*, and *PEX6*, which are associated with fat deposition, were highlighted. This research offers valuable insights into the genetic basis of phenotypic variation among Chinese sheep breeds [76]. In addition, *SPAG17*, *Tbx15*, *VRTN*, *NPC2*, *BMP2*, and *PDGFD* were identified as candidate genes for the tail type characteristics in sheep. Based on these identified tail type trait candidate genes, a SNP (rs69 C>A) was detected in the first exon of the *BMP2* and one SNP (rs69 C>A) in the fourth exon of the *PDGFD*. Interestingly, in Altay sheep, rs119 is the TT genotype; whereas in Tibetan sheep, it is the CC genotype. At the rs69 locus of the *PDGFD*, Altay sheep exhibit the CC genotype; while Tibetan sheep show the AA genotype [77]. Li et al [78] generated a high quality sheep genome assembly using PacBio high fidelity sequencing. They identified 122 SVs that emerged during domestication across 690 sheep breeds globally. Notably, a new 168 bp insertion at a high frequency was detected in the 5’UTR of *HOXB13*. Subsequent GWAS and gene expression analysis demonstrated that this mutation is a key determinant of long tail traits in sheep.

#### Selection signatures analysis

Xu et al [79] genotyped the Han sheep population using the Infinium HD SNP BeadChip. Through GWAS, *CREB1*, *STEAP4*, *CTBP1*, and *RIP140*, genes involved in lipid storage or adipocyte regulation, were identified. Additionally, the OARX: 88–89 Mb region was pinpointed as a strong candidate genomic region for fat deposition in the sheep tail. Furthermore, a selection signature analysis indicated that *HOXA11*, *BMP2*, *PPP1CC*, *SP3*, *SP9*, *WDR92*, *PROKR1*, and *ETAA1* may play crucial roles in fat tail formation [80]. Qi et al [81] utilized two sheep with extreme tail types (Mongolian sheep and Bamei mutton sheep) to analyze genetic differences at the genomic level, aiming to identify candidate genes associated with tail type. Based on Fst, π ratios, and XP-EHH methods, they identified *PDGFD*, *GLIS1*, *AR*, and *FGF9* as genes related to fat deposition in sheep tails, while *VRTN* was associated with tail length and may play a significant role in the formation of sheep tail types. Zhao et al [82] used hapFLK to analyze the selection signature of three breeds of sheep, and identified that *WDR92*, *TBX12*, *WARS2*, *BMP2*, *VEGFA*, *PDGFD*, *HOXA10*, *ALX4* and *ETAA1* genes were related to sheep tail type.

### High altitude adaptation trait

Genetic variation analysis within the putative sweep region revealed the presence of the *SOCS2* gene in native lowland and high altitude adapted Tibetan sheep [78]. Guo et al [28] compared cashmere goat breeds from the Tibetan highlands and lowlands. Through this comparison, they identified different selection targets associated with high altitude adaptation, namely *JUP*, *ICAM1*, *YES1*, and *SIRT1*. The candidate genes *SIRT1* and *YES1* are involved in upregulating the hypoxia inducible factors *HIF2* and *HIF1* respectively. Meanwhile, *ICAM1* is induced in cardiac muscles and aortic endothelial cells under hypoxic conditions. Furthermore, the *EPAS1* gene, which is involved in oxygen sensing and regulating hemoglobin concentration, was detected in the selection signature region associated with high altitude adaptation in Tibetan goats [28]. By applying Fst based DI statistics for population differentiation analysis of Tibetan goats, candidate genes such as *EGFR*, *FGF2*, *ENPEP*, *PTEN*, *AKT1*, *KDR*, *SIRT6*, *CDC42*, and *MITF* were identified. These genes play significant roles in hypoxic conditions and contribute to adaptation to high level UV radiation, energy metabolism, and angiogenesis at high altitudes. Among them, genes identified in the selection region, like *LEPR*, *FGF2*, and *LDB1* in the lungs and *LEPR*, *EGFR*, and *LDB1* in the heart, exhibited higher expression levels. These genes are likely associated with high altitude adaptation and serve as novel selection signals in Tibetan goats [83]. Li et al [84] identified candidate genes *PTEN*, *ATAD1*, and *PAPSS2* in the genomic region of chromosome 26, which are involved in high altitude adaptation in Tibetan goats. In addition, Zhong et al [85] employed the Fst and XP-EHH statistical tools to identify the *ADIRF* as a candidate related to high altitude adaptation in indigenous Chinese goat breeds. Moreover, the *CHL1* was found to be associated with high altitude hypoxic conditions in Youzhou dark goats [32]. Wang et al [21] integrated ZHp and DI values to validate hypoxia adaptation related genes across multiple populations, including *CDK2*, *SOCS2*, *NOXA1*, and *ENPEP*. Exome sequencing further demonstrated that *EPAS1* was enriched specifically in the high altitude cashmere goat population [86].

### Others

**Figure 3:**
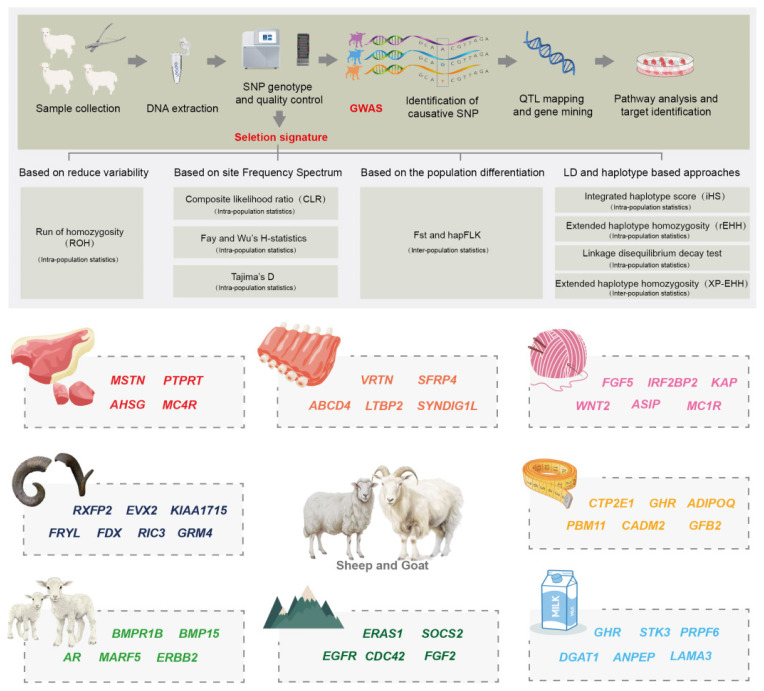
A brief overview of genome wide association studies (GWAS) and selection signature analysis methods and economically and agriculturally important genes in sheep and goat genomes.

Selection signatures were detected across the goat genomic region. Specifically, genes such as *DOCK8*, *IL1R1*, *IL7*, *CD28*, *CD274*, *IL1A*, *TLR2, SLC25A31*, *IRF3*, and *SRSF3* were identified to be associated with the immune response; *PANK2* and *NMNAT2* with visual function; *HBE*, *GF*, *SOSTDC1*, *ARNT*, *COL4A1/2*, and *EP300* with cold tolerance, and *PDE10A* with body temperature regulation [44,66,87]. In dairy goats, *JAK1*, *POU2F2, LRRC66*, *CTSZ*, and *NELFCD* were specifically linked to immunization [67,68]. The GeneSeek Genomic Profiler Goat 70K SNP chips were used for genotyping. GWAS identified *ARHGAP11A* and *GJD2* as key genes related to meat pituitary traits in milk goats. Moreover, GWAS confirmed that the presence of meat pituitary had no significant impact on milk yield [88]. GWAS and Linkage disequilibrium analysis were conducted to precisely map the genetic locus underlying the polled phenotype in goats. During goat domestication, genes such as *STIM1*, *RRM1*, *MUC6*, *IFNAR1*, *FREM3, CD84*, and *NKG2D* underwent strong selection. Through alignments with different reference genomes (T2T-goat1.0 and ARS1), *MUC6*, *GIMAP8*, *CNTNAP2*, *KCND3*, and *NBEAL1* were found to possess immunological activity; *ABCC4*, *PSIP1*, and *TOMM70* were associated with stress resistance; and *SPAG16* was related to sperm flagellar function [19]. Using the publicly available ancient goat genome, Yang et al [43] explored whether common genes were selected at different stages of domestication and early development. They identified a total of five common SV genes (*DNER*, *KANK1*, *MDGA2*, *LRRC36*, and *SCFD2*) that showed significant associations with neurodevelopment and function [12]. The vertebral number is a key economic trait that can significantly influence carcass length and meat yield in animals [89]. By performing selective scanning analysis on sheep resequencing data for multiple lumbar and normal lumbar spine numbers, Li et al [90] found that *SFRP4* was annotated in the 49.68–49.74 MB region of chromosome 4, which was the most highly selective region. To further detect SNPs of the putative candidate *SFRP4*, the PCR-SSCP technology was employed. Two SNPs of this gene (rs600370085:C>T and rs415133338:A>G) were significantly associated with multiple lumbar vertebrae in Duolang sheep. In order to identify candidate genes and QTLs related to sheep vertebrae number, Zhou et al [91] identified *VRTN*, *SYNDIG1L*, *LTBP2* and *ABCD4* as key genes regulating spine development and morphology. It is noteworthy that the mutation in *ABCD4* gene (Chr 7:89393414, C>T) at position 22 caused the conversion of arginine (Arg) to glutamine (Gln), which is expected to negatively affect protein function. Further studies showed that the expression level of *VRTN* gene was significantly higher in sheep with higher thoracic numbers than in sheep with normal thoracic numbers during fetal development. Genome-wide comparison between increased thoracic spine numbers and normal sheep revealed that the *VRTN* gene is the main selection site for thoracic spine numbers in sheep and has the potential for future application in sheep breeding (Figure 3) [92].

## FUTURE CHALLENGES AND PROSPECTS

### Challenges

#### The challenges of deciphering complex genomic structures

Sheep and goat genomes are highly complex, with abundant repetitive sequences, SVs, and polymorphic regions. For example, 40%–50% of the caprine genome consists of repetitive elements, whose tandem repeats vary significantly among individuals and breeds, challenging genome assembly and annotation. This complexity poses analytical hurdles for GWAS and selection signature analyses, increasing false positives in GWAS and masking true selection signals. Take sheep tail morphology: its diverse phenotypes (long, short, fat tails) result from complex gene-network interactions, while repetitive elements and SVs may affect gene expression, complicating genetic dissection via conventional approaches.

#### The dilemma of sample and phenotypic data acquisition

For valid GWAS and selection signature analyses, large-scale representative sampling is crucial. In China, the wide distribution and rich breed diversity of sheep and goats pose sampling challenges, especially for small, fragmented populations like Tibetan sheep in remote high-altitude areas. Genetic divergence among breeds further complicates comprehensive sampling. Precise phenotyping is another hurdle. Assessing economic traits (meat quality, reproductive, milk performance) requires specialized tools and standardized protocols, while environmental factors (feeding, temperature) introduce noise that masks genetic signals, weakening analysis power.

#### Technical bottlenecks in data analysis and processing

Sequencing advances generate massive sheep/goat genomic data, with large-scale GWAS involving millions of SNPs in tens of thousands of samples. This demands huge storage and computing resources, but high costs and bandwidth limits restrict many institutions. Analyzing 1,000 samples with 1M SNPs needs terabytes of storage and hundreds of compute hours. Existing GWAS and selection signal methods have limitations. Linear mixed models in GWAS may miss trait-related loci by ignoring complex genetic/environmental interactions. Selection signal methods (e.g., Fst, π) yield inconsistent results due to differing assumptions, and integrated analysis strategies/tools for complex traits are lacking.

### Prospects

Future studies will be extended to more sheep and goat breeds to improve existing genome maps and resolve complex structures, providing a more accurate reference for GWAS and selection signal analysis to identify genetic variants related to economic traits and adaptability. Moreover, integrating transcriptomics, proteomics, and metabolomics technologies helps analyze gene expression profiles across tissues and developmental stages, constructing a gene regulation network to deepen the understanding of complex traits’ genetic basis. Researchers need to develop new analysis methods, such as multi-effect GWAS models and AI driven genomic mining, while strengthening international cooperation to share data and resources for joint research on sheep and goat genetic resources assessment and utilization.

## CONCLUSION

This review highlights progress in GWAS and selection signal analysis of economically important traits in Chinese sheep and goats, identifying key genomic regions and candidate genes (e.g., *FGF5* for wool trait, *BMPR1B* for reproduction trait). Challenges remain in refining reference genomes, phenotypic data collection, algorithm optimization, functional validation, and translational research. Future directions include genome improvement, multi-omics integration, algorithm development, and international collaboration to decode genetic mechanisms, enabling precise marker-assisted selection for global sheep/goat industry sustainability.

## Figures and Tables

**Table 1:** Reference genomes available in NCBI for sheep and goat

Species	Release	Genome assembly	Genome size (Gb)	Sequencing centre	Breeds
Sheep (*Ovis aries*)	2010	Ovis_aries_1.0	2.9	International Sheep Genomics Consortium	Mixed
	2015	OARv4.0	2.71		Texel
	2017	Oar_rambouillet_v1.0	2.71	Baylor College of Medicine Human Genome Sequencing Center	Rambouillet
	2021	CAU_O.aries_1.0	2.7	China Agricultural University	Tibetan sheep
	2023	ARS-UI_Ramb_v3.0	2.7	University of Idaho	Rambouillet
	2024	T2T-sheep1.0	2.9	China Agricultural University	Hu sheep
Goat (*Capra hircus*)	2015	CHIR_2.0	2.8	International Goat Genome Consortium, Beijing Genomics Institute	Yunnan black goat
	2016	ARS1.2	2.9	USDA ARS	San Clemente
	2020	Saanen_v1	2.7	Northwest A&F University	Saanen dairy goat
	2024	T2T-goat1.0	2.9	China Agricultural University	Inner Mongolia cashmere goat
	2024	T2T-goat2.0	2.9		

## Data Availability

Upon reasonable request, the datasets of this study can be available from the corresponding author.
